# Pregnancy-associated progression of chronic kidney disease: a study protocol for the development and validation of a clinical predictive tool (PREDICT)

**DOI:** 10.1007/s40620-023-01788-5

**Published:** 2023-11-21

**Authors:** Elizabeth Ralston, Michelle Hladunewich, Chris Farmer, Juan-Jesus Carrero, PREDICT Investigation Group, Kate Wiles, Yanzhong Wang, Amanda Clery, Joseph Chilcot, Steve Childs, Yuanhang Yang, Nivethika Jeyakumar, Amit Garg, Lavanya Bathini, Graham Smith, Hannah Blakey, Nadia Sarween, Graham Lipkin, Ellen Knox, Tess Harris, David Pitcher, Shalini Santhakumaran, Maria Casula, Retha Steenkamp, Lucy Chappell, Philip Webster, Sue Carr, Matthew Hall, Liz Lightstone, Kate Bramham, Kate Bramham

**Affiliations:** 1 King’s College London, 5th Floor Addison House, Guy’s Campus, London, SE1 1UL UK; 2 Division of Nephrology, University Health Network and University of Toronto, Toronto, Canada; 3 Centre for Health Services Studies, University of Kent, Kent, UK; 4 Department of Medical Epidemiology and Biostatistics, Karolinska Institutet, Stockholm, Sweden

Women with chronic kidney disease (CKD) in reproductive years may frequently wish to consider the option of pregnancy. It is estimated that early-stage CKD is present in 3:100 pregnancies, and later stage CKD affects 1:750 pregnancies [1]. Chronic kidney disease is associated with an increased risk of maternal and neonatal adverse outcomes which are inversely related to deteriorating kidney function and include pre-eclampsia, small-for-gestational-age infants, preterm birth, and accelerated decline in kidney function postpartum [2]. There is no reliable contemporaneous prediction tool for pregnancy and kidney outcomes for women with CKD, despite requests from women with CKD and their health care professionals to provide more resources and support [2]. This study aims to develop and validate two prediction models that estimate (i) the likelihood of a ≥ 25% reduction in estimated glomerular filtration rate (eGFR) or initiation of renal replacement therapy between 6 weeks and 12 months postpartum and (ii) the likelihood of a small-for-gestational-age (SGA < 3rd percentile) infant and/or premature birth < 34 weeks’ gestation.

The models will be developed and validated using seven international datasets. Model development will use the National Registry of Rare Kidney Diseases (RaDaR) from the UK Renal Registry (UKRR), a United Kingdom (UK) wide, linked dataset between RaDaR, UKRR, Hospital Episode Statistics (HES) and Maternity Services Data Set (MSDS). This cohort includes any woman with a diagnosis of kidney disease in the UK with maternity records from 1997 onwards, including those with ICD-10 codes for CKD in Hospital Episode Statistics, consented to participate in RaDaR or with data reported to UKRR.

External validation will be performed in the following datasets:

i. West and East Kent integrated maternal and laboratory data from the University of Kent. This includes routinely collected data from hospitals in Kent (UK).
ii. Stockholm Creatinine Measurement (SCREAM). This is an observational dataset with laboratory data of individuals residing or accessing healthcare in the region of Stockholm (Sweden) with results. SCREAM has previously been described in detail [3]
iii. The Ontario Renal Network Pregnancy Cohort. This is a population-based cohort of women in the province of Ontario (Canada) with a baby born between 2007 and 2020. Administrative health databases linked using unique identifiers at Institute for Clinical Evaluative Sciences (ICES) are used to capture all hospital births in Ontario and outpatient laboratory testing.
iv. Cohort data from three obstetric studies within the UK collected between 2010 and 2018. These studies have previously been described in detail [2, 4, 5].

The model development and validation will follow the Transparent Reporting of a multivariable prediction model for Individual Prognosis Or Diagnosis statement recommendations (TRIPOD) [6]. Ethical approval was given by the Health Research Authority (London Bloomsbury Research Ethics Committee, Ref: 23/LO/0258). The study is registered on ClinicalTrials.gov (Ref: NCT05793346).

Women with an eGFR less than 90 ml/min/1.73 m^2^ using the Chronic Kidney Disease Epidemiology Collaboration (CKD-EPI 2009) [7] without ethnicity adjustment within 24 months prior to conception will be included. Women established on dialysis at time of conception, multi-fetal pregnancies, known inpatient eGFR measurement and those with no preconception eGFR within 24 months will be excluded.

Candidate model predictors are based on previous studies reporting risk factors for pregnancy and kidney outcomes in women with CKD and availability within the cohorts (Table 1). All predictors are within routine care for women with CKD in pregnancy enabling the acceptability of implementation and generalisability of the models.

**Table 1:** Candidate predictors for model development

Candidate predictor	Definition	Measurement unit
Maternal age	Age at conception	Years
Maternal ethnicity	Self-reported ethnicity. This, if possible is categorised into four groups following published guidance from UK government	Asian, Black, Caribbean or African, mixed or multiple ethnic groups, White and other
Maternal BMI	Maternal body mass index (BMI) at first pregnancy visit	kg/m^2^
Multiparous	Prior record of delivery after 24 weeks’ irrespective of outcome	Yes or no
Chronic hypertension	Antihypertensive medications before pregnancy or before 20 weeks’ gestation/or systolic blood pressure > 140 mmHg or diastolic blood pressure > 90 mmHg before 20 weeks’ gestation, or ICD 10 code for hypertension, or two physician claims for hypertension within two years prior to pregnancy	Yes or no
Aetiology of CKD during pregnancy	Primary cause of CKD categorised into eight pre-defined categories. (1) glomerulonephritis (excluding systemic lupus erythematosus), (2) chronic Pyelonephritis or Vesicoureteral reflux, (3) autosomal dominant polycystic kidney disease (ADPKD), (4) diabetic nephropathy (not including women with other CKD cause and co-existing diabetes), (5) congenital or inherited (other than ADPKD), (6) kidney transplant, (7) systemic lupus erythematosus, (8) other	
Pre pregnancy eGFR	eGFR within 24 months of conception calculated using CKD-EPI equation without ethnicity adjustment	ml/min/1.73 m^2^
Pre pregnancy proteinuria	uPCR, uACR or urine dipstick within 24 months of conception	Normal (uACR < 3 or uPCR < 15 mg/mmol, urine dipstick normal) moderate (uACR 3–30, uPCR 15–50 mg/mmol, or urine dipstick 1 +). severe (uACR > 30, uPCR > 50 mg/mmol or urine dipstick 2 +)

*CKD* chronic kidney disease, *CKD-EPI* Chronic Kidney Disease Epidemiology Collaboration 2009, *eGFR* estimated glomerular filtration rate, *ICD* International Classification of Diseases, *uACR* urine albumin-to-creatinine ratio, *uPCR* urine protein-to-creatinine ratio

There are two binary outcome measures. Firstly, a ≥ 25% reduction in eGFR or initiation of renal replacement therapy between 6 weeks and 12 months postpartum. This timeframe is applied as typically, serum creatinine levels may peak within the first few weeks postpartum but return to pre-pregnancy concentrations [8]. This outcome was chosen from surveys including 90 women with CKD and 73 healthcare professionals. The second outcome is a composite outcome of preterm birth defined as less than 34 weeks’ gestation and/or SGA < 3rd percentile (INTERGROW).

This is a secondary analysis on pre-existing data and therefore the sample size is fixed; however using *pmpsampsize* package, a sample size of at least 494 pregnancies is estimated to be adequate to avoid overfitting [9]. Characteristics of the cohort for model development and validation will be described, including missing data. Listwise deletion will be applied for model development. A sensitivity analysis will be performed using multiple imputation.

The development of both models will follow the same analyses as both outcomes are dichotomous. Initial univariable logistic regression models will be performed on each candidate predictor to initially assess their crude association with the outcome and we will fit a multivariable model containing all predictors. Then backwards elimination will be used to successively remove the least significant predictors in the model using the Akaike’s Information Criterion and significance. A liberal *p* value of 0.10 will be applied for retention. We will also consider predictors based on clinical knowledge and previous research findings. Variance inflation factor will be performed prior to fitting the final model to identify any collinearity between the predictor variables. The final predictors will be included in a multivariable logistic regression model. The final models will be assessed for overall performance (model fit), calibration and discrimination. Clinical performance will be assessed through positive predictive value, negative predictive value, sensitivity and specificity.

Final models will be subject to internal and external validation to assess performance of the models. Internal validation using bootstrapping will enable us to examine for potential overfitting of the developed models. We will externally validate the final models in the international cohorts. The overall predictive performance, clinical performance, discrimination and calibration will be evaluated in each cohort.

The purpose of this study is to develop and validate two prediction models estimating the likelihood of: (i) having a 25% or greater decline in kidney function or initiation of renal replacement therapy, and (ii) having a delivery before 34 weeks or a SGA infant. The provision of these models will help to facilitate informed decision-making amongst women and their partners. The provision of risk information will support clinicians in providing personalised maternity counselling and care.

## References

[1] Piccoli G, Zakharova E, Attini R (2018). Pregnancy in chronic kidney disease: need for higher awareness. A pragmatic review focused on what could be improved in the different CKD stages and phases. J Clin Med.

[2] Wiles K, Webster P, Seed PT (2020). The impact of chronic kidney disease stages 3–5 on pregnancy outcomes. Nephrol Dialysis Transplant.

[3] Carrero JJ, Elinder CG (2022). The Stockholm CREAtinine Measurements (SCREAM) project: fostering improvements in chronic kidney disease care. J Intern Med.

[4] Bramham K, Seed PT, Lightstone L (2016). Diagnostic and predictive biomarkers for pre-eclampsia in patients with established hypertension and chronic kidney disease. Kidney Int.

[5] Sarween N, Hodson J, Knox E (2019). A study of maternal and fetal outcomes in a cohort of pregnant women with chronic kidney disease. Nephrol Dialysis Transplant.

[6] Moons KGM, Altman DG, Reitsma JB (2015). Transparent reporting of a multivariable prediction model for individual prognosis or diagnosis (TRIPOD): explanation and elaboration. Ann Intern Med.

[7] Levey A, Stevens L, Schmid C (2009). A new equation to estimate glomerular filtration rate. Ann Intern Med.

[8] Harel Z, McArthur E, Hladunewich M (2019). Serum creatinine levels before, during, and after pregnancy. JAMA J Am Med Assoc.

[9] Ensor J, Martin E, Riley R (2021) Pmsampsize: calculates the minimum sample size required for developing a multivariable prediction model. https://cran.r-hub.io/web/packages/pmsampsize/pmsampsize.pdf

